# Rationale and Plan for Vitamin D Food Fortification: A Review and Guidance Paper

**DOI:** 10.3389/fendo.2018.00373

**Published:** 2018-07-17

**Authors:** Stefan Pilz, Winfried März, Kevin D. Cashman, Mairead E. Kiely, Susan J. Whiting, Michael F. Holick, William B. Grant, Pawel Pludowski, Mickael Hiligsmann, Christian Trummer, Verena Schwetz, Elisabeth Lerchbaum, Marlene Pandis, Andreas Tomaschitz, Martin R. Grübler, Martin Gaksch, Nicolas Verheyen, Bruce W. Hollis, Lars Rejnmark, Spyridon N. Karras, Andreas Hahn, Heike A. Bischoff-Ferrari, Jörg Reichrath, Rolf Jorde, Ibrahim Elmadfa, Reinhold Vieth, Robert Scragg, Mona S. Calvo, Natasja M. van Schoor, Roger Bouillon, Paul Lips, Suvi T. Itkonen, Adrian R. Martineau, Christel Lamberg-Allardt, Armin Zittermann

**Affiliations:** ^1^Division of Endocrinology and Diabetology, Department of Internal Medicine, Medical University of Graz, Graz, Austria; ^2^Clinical Institute of Medical and Chemical Laboratory Diagnostics, Medical University of Graz, Graz, Austria; ^3^Synlab Academy, Synlab Services GmbH, Mannheim, Germany; ^4^Cork Centre for Vitamin D and Nutrition Research, School of Food and Nutritional Sciences, University College Cork, Cork, Ireland; ^5^College of Pharmacy and Nutrition, University of Saskatchewan, Saskatoon, SK, Canada; ^6^Section of Endocrinology, Nutrition and Diabetes, Department of Medicine, Physiology and Biophysics, Boston University Medical Center, Boston, MA, United States; ^7^Sunlight, Nutrition and Health Research Center, San Francisco, CA, United States; ^8^Department of Biochemistry, Radioimmunology and Experimental Medicine, The Children's Memorial Health Institute, Warsaw, Poland; ^9^Department of Health Services Research, CAPHRI Care and Public Health Research Institute, Maastricht University, Maastricht, Netherlands; ^10^Bad Gleichenberg Clinic, Bad Gleichenberg, Austria; ^11^Department of Cardiology, Swiss Cardiovascular Center Bern, Bern University Hospital, Bern, Switzerland; ^12^Department of Laboratory Medicine, Paracelsus Medical University, Salzburg, Austria; ^13^Division of Cardiology, Department of Internal Medicine, Medical University of Graz, Graz, Austria; ^14^Department of Pediatrics, Medical University of South Carolina, Charleston, SC, United States; ^15^Department of Endocrinology and Internal Medicine, Aarhus University Hospital, Aarhus, Denmark; ^16^Division of Endocrinology and Metabolism, First Department of Internal Medicine, Medical School, Aristotle University of Thessaloniki, AHEPA Hospital, Thessaloniki, Greece; ^17^Institute of Food Science and Human Nutrition, Leibniz University Hannover, Hannover, Germany; ^18^Department of Geriatrics and Aging Research, University Hospital Zurich and Waid City Hospital, University of Zurich, Zurich, Switzerland; ^19^Center for Clinical and Experimental Photodermatology, The Saarland University Hospital, Homburg, Germany; ^20^Tromsø Endocrine Research Group, Department of Clinical Medicine, UiT The Arctic University of Norway, Tromsø, Norway; ^21^Department of Nutritional Sciences, Faculty of Life Sciences, University of Vienna, Vienna, Austria; ^22^Department of Nutritional Sciences, University of Toronto, Toronto, ON, Canada; ^23^School of Population Health, University of Auckland, Auckland, New Zealand; ^24^U.S. Food and Drug Administration, Silver Spring, MD, United States; ^25^Department of Epidemiology and Biostatistics, Amsterdam Public Health Research Institute, VU University Medical Center, Amsterdam, Netherlands; ^26^Laboratory of Clinical and Experimental Endocrinology, Department of Chronic Diseases, Metabolism and Ageing, KU Leuven, Leuven, Belgium; ^27^Endocrine Section, Department of Internal Medicine, VU University Medical Center, Amsterdam, Netherlands; ^28^Calcium Research Unit, Department of Food and Nutrition, University of Helsinki, Helsinki, Finland; ^29^Barts and The London School of Medicine and Dentistry, Queen Mary University of London, London, United Kingdom; ^30^Clinic for Thoracic and Cardiovascular Surgery, Heart Center North Rhine-Westfalia, Ruhr University Bochum, Bad Oeynhausen, Germany

**Keywords:** vitamin D, public health, food fortification, general population, guidelines, evidence, recommendations, policy

## Abstract

Vitamin D deficiency can lead to musculoskeletal diseases such as rickets and osteomalacia, but vitamin D supplementation may also prevent extraskeletal diseases such as respiratory tract infections, asthma exacerbations, pregnancy complications and premature deaths. Vitamin D has a unique metabolism as it is mainly obtained through synthesis in the skin under the influence of sunlight (i.e., ultraviolet-B radiation) whereas intake by nutrition traditionally plays a relatively minor role. Dietary guidelines for vitamin D are based on a consensus that serum 25-hydroxyvitamin D (25[OH]D) concentrations are used to assess vitamin D status, with the recommended target concentrations ranging from ≥25 to ≥50 nmol/L (≥10–≥20 ng/mL), corresponding to a daily vitamin D intake of 10 to 20 μg (400–800 international units). Most populations fail to meet these recommended dietary vitamin D requirements. In Europe, 25(OH)D concentrations <30 nmol/L (12 ng/mL) and <50 nmol/L (20 ng/mL) are present in 13.0 and 40.4% of the general population, respectively. This substantial gap between officially recommended dietary reference intakes for vitamin D and the high prevalence of vitamin D deficiency in the general population requires action from health authorities. Promotion of a healthier lifestyle with more outdoor activities and optimal nutrition are definitely warranted but will not erase vitamin D deficiency and must, in the case of sunlight exposure, be well balanced with regard to potential adverse effects such as skin cancer. Intake of vitamin D supplements is limited by relatively poor adherence (in particular in individuals with low-socioeconomic status) and potential for overdosing. Systematic vitamin D food fortification is, however, an effective approach to improve vitamin D status in the general population, and this has already been introduced by countries such as the US, Canada, India, and Finland. Recent advances in our knowledge on the safety of vitamin D treatment, the dose-response relationship of vitamin D intake and 25(OH)D levels, as well as data on the effectiveness of vitamin D fortification in countries such as Finland provide a solid basis to introduce and modify vitamin D food fortification in order to improve public health with this likewise cost-effective approach.

## Introduction

Vitamin D deficiency is common worldwide and potential adverse effects of a poor vitamin D status are of concern for public health (1–4). In this review, we aim to provide an overview on the rationale, current status and implementation plans for vitamin D food fortification as a means to close the gap between widespread inadequate vitamin D intakes and the target vitamin D intakes as recommended by nutritional vitamin D guidelines (4–8). This work should ideally provide a basis for the communication with and guidance for health authorities and regulators that are responsible for food policy and potential food fortification within their respective countries or regions.

This paper is based on a systematic literature search in PubMed until the end of March 2018 using the search terms “vitamin D” and “fortification,” but reference lists of retrieved articles and personal references were also used. After an introduction on metabolism and clinical effects of vitamin D, we briefly summarize major nutritional vitamin D guidelines and give an overview on global vitamin D status and vitamin D intakes with a focus on the gap that exists between current estimates for vitamin D requirements and actual vitamin D intakes within populations. Following a section on general approaches on how to prevent and treat vitamin D deficiency, we outline safety issues of vitamin D before we present data on the history and the current status of vitamin D food fortification world-wide. Then, we briefly summarize the approaches and modeling as well as cost-effectiveness studies of vitamin D food fortification. Finally, we present some suggestions and guidance on how to implement vitamin D food fortification.

## Metabolism of Vitamin D

Vitamin D has a unique metabolism and is mainly produced in the skin where exposure to ultraviolet-B (UV-B) radiation (in sunlight) induces the conversion of skin produced 7-dehydrocholesterol into vitamin D_3_ (cholecalciferol) (9). Dietary intake of vitamin D from natural foods traditionally plays only a minor role with few available natural sources: animal sources such as fatty fish, cod liver oil, or egg yolks contain vitamin D_3_, and fungal sources such as mushrooms and yeast exposed to sunlight or UV radiation contain vitamin D_2_ (ergocalciferol). Vitamin D_3_ and D_2_ share, in general, the same metabolism. Therefore, we will not differentiate between these two forms unless otherwise stated and refer to vitamin D (meaning vitamin D_3_ and/or vitamin D_2_) throughout this manuscript. In terms of sources, vitamin D can also be supplied by supplements and fortified foods but vitamin D and its metabolites may also be stored and released from the body‘s adipose tissue (9–11). A very rough general estimate is that about 80% of vitamin D supply comes from UV-B induced production in the skin and about 20% from dietary intake, but this varies considerably depending on factors such as season/sun exposure habits, latitude, nutrition/supplement intake or ethnicity (3, 9, 12). Despite a high degree of inheritance of serum 25-hydroxyvitamin D (25[OH]D) in twin studies, data from a Genome Wide Association Study (GWAS) indicate that serum 25(OH)D concentrations have only a modest overall heritability due to common GWAS single nucleotide polymorphisms (SNPs) of 7.5%, highlighting the great impact of non-genetic factors to the variability in serum 25(OH)D concentrations (13, 14).

Vitamin D itself does not exert significant genomic biological effects and has to be metabolized (9). The common metabolism of vitamin D from any source involves, as a first step, the conversion to 25(OH)D in the liver that is mediated by different 25-hydroxylase enzymes (9). Serum 25(OH)D is the main circulating vitamin D metabolite that is considered to best indicate overall vitamin D status as it reflects vitamin D supply from diverse sources. Serum 25(OH)D has a traced half-life of approximately 2–3 weeks, whereas vitamin D itself has a half-life of only 1 day. In the bloodstream, approximately 85 to 90% of 25(OH)D is bound to vitamin D binding protein (DBP) and 10 to 15% is bound to albumin, so that less than 1% of serum 25(OH)D is unbound or free (15). The classification of vitamin D status is currently based on total serum 25(OH)D concentrations, i.e., the sum of bound and free fractions of both 25(OH)D_2_ and 25(OH)D_3_. It should, however, be acknowledged that there is some discussion regarding whether measuring free 25(OH)D concentrations may also be useful (15, 16). Such considerations are based on the fact that free 25(OH)D may cross the plasma membrane due to its lipophilic properties, whereas only a few organs that are crucial for vitamin D effects such as the kidneys, the parathyroid glands and the placenta are able to take up DBP-bound vitamin D metabolites through endocytosis by the megalin/cubilin complex (15, 16). While this is an active scientific debate, it is well established that 25(OH)D *per se* is hardly biologically active and has to undergo a further hydroxylation step that takes mainly place in the kidneys. In detail, renal 1-alpha-hydroxylase (*CYP27B1)* converts 25(OH)D to 1,25-dihydroxyvitamin D (1,25[OH]2D) that is also called “calcitriol” or the “active vitamin D hormone.” Whereas the rate of 25-hydroxylation in the liver is mainly substrate dependent until a plateau is reached at high serum 25(OH)D concentrations, 1-alpha hydroxylation in the kidneys is under tight control by calcium and phosphate metabolism including parathyroid hormone (PTH), which stimulates 1-alpha-hydroxylation and fibroblast growth factor-23 (FGF-23), which inhibits it.

From a physiological perspective, 1,25(OH)2D functions like a classic steroid hormone (similar to sex or thyroid hormones): after binding of 1,25(OH)2D to the vitamin D receptor (VDR), this complex translocates to the cell nucleus and regulates the expression of hundreds of genes by interacting with its vitamin D responsive elements on the DNA. Whereas serum 1,25(OH)2D levels mainly derive from the kidneys and therefore exert classic endocrine functions, there is also a wide expression of extrarenal 1-alpha-hydroxylase that converts 25(OH)D to 1,25(OH)2D on a local/tissue level thereby contributing to autocrine and paracrine functions of 1,25(OH)2D. Importantly, the expression of VDR in almost all human tissues provides a sound scientific basis to postulate that vitamin D is important for overall human health. Further metabolism and degradation of vitamin D metabolites is initiated by 24-hydroxylase (*CYP24A1*), and after additional hydroxylation and oxidation steps, the resulting water soluble metabolites, one of which is calcitroic acid, are finally excreted in the bile and urine. For a more detailed description of vitamin D metabolism, we refer the reader to more detailed reviews on this topic (9, 15, 17).

## Clinical effects of Vitamin D

Vitamin D is historically known as a substance that can prevent and treat nutritional rickets and osteomalacia (18–20). Rickets is a bone disease that is associated with low serum calcium and low serum phosphate, and is characterized by widening and delay of mineralization of growth plates in bones (18–20). The clinical presentation of rickets includes heterogeneous skeletal and non-skeletal manifestations such as bowing deformities of the bones, development delay or widening of joints (18–20). Severe cases of rickets can lead to hypocalcemic complications including tetany and seizures as well as dilated cardiomyopathy which can be fatal (18–20). Whereas rickets can only occur in open growth plates, osteomalacia constitutes defective mineralization of existing bone (closed growth plates) (18–20). Rickets and osteomalacia can lead to bone deformation (e.g., pelvic deformities in girls with risk of obstructed labor), as well as isolated and global bone pain and muscle weakness (18–20). Apart from rickets and osteomalacia, vitamin D supplementation may prevent falls and fractures in older individuals at risk of vitamin D deficiency, but data from randomized controlled trials (RCTs) on this topic are inconsistent (21–27). This may be explained by different dosing regimens with daily dosing may be beneficial and large intermittent bolus dosing may be detrimental (21–27). Moreover, it is sometimes difficult to disentangle separate effects of vitamin D and calcium, as there exist interactions between them. Several RCTs with a significant benefit used a combined supplementation of calcium plus vitamin D at doses of 17.5–20 μg (700–800 international units, IU) per day (21–27).

**Figure 1:**
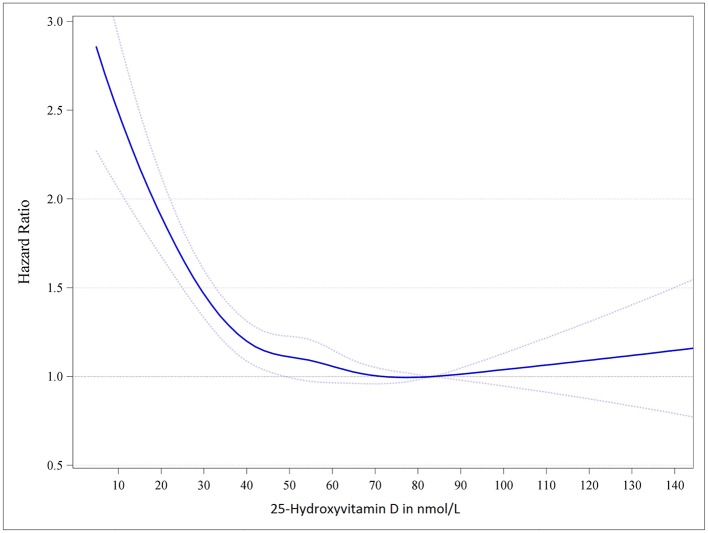
Dose-response trend of hazard ratios of death from all causes by standardized serum 25-hydroxyvitamin D. Dose-response trend of hazard ratios of all-cause mortality by standardized 25-hydroxyvitamin D were adjusted for age, sex, body mass index and season of blood drawing concentrations. Hazard ratios (blue line with 95% confidence intervals as dotted blue lines) are referring to the 25-hydroxyvitamin D concentration of 83.4 nmol/L (i.e., the median 25-hydroxyvitamin D concentration of the group with 25-hydroxyvitamin D concentration from 75 to 99.99 nmol/L). Adopted from Gaksch et al. (28).

Apart from skeletal effects, vitamin D may also have an impact on extra-skeletal health (3, 28–31). Several epidemiological studies have shown that low serum 25(OH)D concentrations are a risk marker for various diseases as well as mortality (3, 28–31) (see Figure 1 for the association between serum 25(OH)D and mortality). Data from meta-analyses of RCTs suggest that vitamin D supplementation may reduce mortality, respiratory tract infections, asthma exacerbations and pregnancy complications, but more data are required to clearly establish causality and doses-response relationships (32–41). Of particular importance are the RCT data suggesting that vitamin D supplementation during pregnancy may be useful in preventing general complications of pregnancy or infant outcomes such as asthma/wheeze (42, 43).

## Nutritional Vitamin D guidelines

Recommendations relating to dietary vitamin D requirements in general populations are termed dietary reference intakes (DRI) or dietary reference values (DRV) (5, 6). These are based on the assumptions that total 25(OH)D serum concentrations are a biomarker of vitamin D status and indicate vitamin D intakes in the absence of cutaneous vitamin D production, which is especially the case in winter at northern latitudes (i.e., in regions far away from the equator). The rationale for nutritional vitamin D recommendations is the establishment of a cause and effect relationship between vitamin D intake and specified health outcomes. To date, vitamin D guidelines have generally been based on beneficial effects of vitamin D on musculoskeletal health outcomes (e.g., rickets, osteomalacia, fractures, muscle weakness, falls etc.) and occasionally on extraskeletal health outcomes such as pregnancy-related health outcomes or mortality. The dose-response relationship is then usually characterized by the association between serum 25(OH)D concentrations and these health outcomes.

As part of this process, certain target concentrations for serum 25(OH)D are established that are then used to calculate the vitamin D intakes for the estimated average requirement (EAR), that is the vitamin D intake at the estimated median requirement, and the recommended dietary allowance (RDA), that is the vitamin D intake that meets or exceeds the vitamin D requirements of 97.5% of the population. If the evidence is insufficient to define a RDA, an adequate intake (AI), is defined. The AI is the recommended average daily intake level of a nutrient based on observed or experimentally determined approximations or estimates of intakes that are assumed to be adequate for a group of apparently healthy people. After setting the target serum 25(OH)D concentrations for the EAR/RDA/AI, the vitamin D intakes that are required to achieve these concentrations thresholds, under circumstances of minimal to no UV-B induced cutaneous vitamin D production, are estimated by meta-regression analyses. The DRV/DRI also assume that the requirements for other nutrients such as e.g., calcium are met. In reality, this is usually not always the case, and vitamin D requirements may therefore even be higher in individuals with inadequate calcium intake, and may also vary according to other factors such as body mass index, ethnicity or genetic polymorphisms related to vitamin D metabolism/effects (13, 44).

An excellent overview of nutritional vitamin D guidelines is published elsewhere (5). For the US and Canada, the Institute of Medicine (IOM) report on vitamin D and calcium was released in 2010 and is considered the benchmark for nutritional vitamin D guidelines (45, 46). The IOM DRI report together with the European Food Safety Authority (EFSA) DRV report can be regarded as the main nutritional vitamin D guidelines (45–47). Therefore, we list the DRV/DRI of these two main guidelines together with three of the, in our opinion, most relevant national guidelines [i.e., Scientific Advisory Committee on Nutrition (SACN)report from the UK, the report from the Nutritional Societies in Germany Austria and Switzerland (DACH) and those of the Nordic European countries (NORDEN)] in Table 1 (45–50). These recommendations are based on conditions of minimal or no endogenous vitamin D synthesis. Apart from these nutritional vitamin D guidelines for the general healthy population, there are also vitamin D guidelines or recommendations published that aim to guide vitamin D diagnostics and supplementation in patients or specific groups, an issue that is beyond the scope of the current article (51–53). A few of these guidelines recommend relatively high target serum 25(OH)D concentrations such as 75 nmol/L (divide by 2.496 to convert nmol/L to ng/mL) because for some musculoskeletal health outcomes and parameters of mineral metabolism such as PTH, these levels may be optimal, whereas target serum 25(OH)D concentrations for effects on the immune system are not clearly established but may even be higher (3, 51, 53).

**Table 1:** Dietary reference values (DRV)/dietary reference intakes (DRI) for vitamin D.

**Country (health authority)**	**USA and Canada (IOM)**		**Europe (EFSA)**	**Germany, Austria and Switzerland (DACH)**	**UK (SACN)**	**Nordic European countries (NORDEN)**
**DRV/DRI**	**EAR**	**RDA**	**AI**	**AI**	**RNI**	**RI**
**Target 25(OH)D in nmol/L**	**40**	**50**	**50**	**50**	**25**	**50**
**Age group**	**Vitamin D intakes in** μ**g per day (1** μ**g** = **40 international units)**					
0–6 months	10			10	8.5–10	
7–12 months	10		10	10	8.5–10	10
1–3 years	10	15	15	20	10	10
4–6 years	10	15	15	20	10	10
7–8 years	10	15	15	20	10	10
9–10 years	10	15	15	20	10	10
11–14 years	10	15	15	20	10	10
15–17 years	10	15	15	20	10	10
18–69 years	10	15	15	20	10	10
70–74 years	10	20	15	20	10	10
75 years and older	10	20	15	20	10	20
Pregnancy	10	15	15	20	10	10
Lactation	10	15	15	20	10	10

*IOM, Institute of Medicine; EFSA, European Food Safety Authority; DACH, Germany, Austria and Switzerland; SACN, Scientific Advisory Committee on Nutrition; EAR, Estimated Average Requirement; RDA, Recommended Dietary Allowance; AI, Adequate Intake; RNI, Reference Nutrient Intake; RI, Recommended; Intake; 25(OH)D, 25-hydroxyvitamin D*.

## Global Vitamin D status and Vitamin D intakes

Several studies have investigated the prevalence of low serum 25(OH)D concentrations and of inadequate vitamin D intakes in general populations worldwide (54–63). It is obvious from these various reports that serum 25(OH)D concentrations and vitamin D supply are insufficient to meet the vitamin D requirements in significant sections of the general population worldwide. There exist, of course, regional differences in the burden of vitamin D deficiency, but it can be clearly stated that vitamin D deficiency is a worldwide public health problem. According to recent surveys, serum 25(OH)D concentrations <30 nmol/L and <50 nmol/L are documented in 13.0 and 40.4% of the general population in Europe, and in 6.7 and 26.0% of the general population in the US, respectively (55, 57, 58). Compared to Europe and North America, the prevalence of low serum 25(OH)D concentrations seems to be even higher in many low and lower-middle income countries (56). In India, Tunisia and Mongolia, for example, the prevalence of serum 25(OH)D concentrations below 25/30 nmol/L exceeds 20% in the entire population (56). It should be noted that interpretation of some previous vitamin D status data may be limited due to differences/problems of laboratory assays, whereas many recent surveys were based on well standardized 25(OH)D measurements (55).

Data on dietary vitamin D intakes are less complete compared to data on serum 25(OH)D concentrations, but it can be generally stated that in the majority of the countries worldwide, the median vitamin D intake is below 5 μg (200 IU) per day (62). It should, however, be acknowledged that assessment of vitamin D intakes is not trivial because food composition data are not always up-to-date with regard to actual vitamin D content of food. Furthermore, 25(OH)D content of food has often not been considered although this plays a significant role for vitamin D status in consideration of the fact that 25(OH)D_3_ is approximately 5 times as effective as an equivalent intake of vitamin D_3_ in terms of increasing serum 25(OH)D concentrations (4).

## Approaches to prevention and treatment of Vitamin D deficiency

Approaches to improve vitamin D status in the population include increasing intake of naturally vitamin D containing food, food fortification, vitamin D supplements, increasing solar UV-B exposure and weight loss (64–76). Promotion of weight loss which may mobilize vitamin D and its metabolites from the adipose tissue as well as increasing intake of naturally vitamin D containing food (e.g., fatty fish) can be considered as general steps toward a healthier lifestyle but such attempts have usually an insufficient overall impact on vitamin D status. Nevertheless, a meta-analyses of RCTs showed that compared to controls, fish consumption, which is usually the highest food source of vitamin D, raised serum 25(OH)D concentrations on average by 4.4 nmol/L (75). Recommendations regarding more sunlight (UV-B) exposure have the potential to increase serum 25(OH)D concentrations but are limited by adverse effects related to skin damage and skin cancer. Use of vitamin D supplements represents an effective strategy for the prevention and treatment of vitamin D deficiency at the individual level, but adherence within the general population as well as potential overdosing of vitamin D supplements are significant limitations. In the US, 3.2% in the general population take vitamin D supplements at a dose of ≥100 μg (4,000 IU) per day (10). It should also be underlined that supplement intake positively correlates with a healthier lifestyle and higher socio-economic status suggesting that recommendations for supplement intake do not adequately reach those people at particular high risk of vitamin D deficiency.

Therefore, vitamin D food fortification seems to be the most appropriate way of improving vitamin D intake and status in the general population in order to meet dietary vitamin D recommendations. In general, food can be enriched with vitamin D by simply adding vitamin D to food (i.e., traditional vitamin D food fortification) or by so called “bioaddition.” Bioaddition of vitamin D, which has also been called “biofortification,” refers to various ways of increasing vitamin D content of food without direct exogenous addition of vitamin D. Examples of bioaddition include feeding hens with vitamin D (and/or 25[OH]D) to increase the vitamin D (and/or 25[OH]D) content of the eggs, increasing vitamin D content of feed for farmed fish to increase their flesh vitamin D content, likewise with livestock animals in relation to meat, and UV exposure of mushrooms or yeast (that is then used to make bread), which facilitates the conversion of ergosterol to vitamin D_2_. These issues are discussed in detail elsewhere (4, 74).

## Safety issues for Vitamin D

When discussing public health strategies to increase vitamin D intakes in the general population, the potential dual harm of both deficiency and excess of vitamin D must be considered (77–83). Large oral doses of vitamin D increase serum 25(OH)D concentrations while serum 1,25(OH)2D concentrations are usually not materially changed and can even be reduced (79). It has been hypothesized that at very high serum 25(OH)D concentrations the binding capacity of the DBP may be exceeded leading to a release of free and biologically active vitamin D metabolites. Clinically, vitamin D intoxication can lead to hypercalciuria which precedes hypercalcemia. Consequences of hypercalciuria may include the formation of kidney stones, nephrocalcinosis and reduced kidney function. Hypercalcemia can be associated with fatigue, muscle weakness, weight loss, nausea, vomiting, soft tissue calcification or tachycardia. Recent RCTs using relatively high vitamin D doses have significantly increased our knowledge on the safety of vitamin D treatment (84–91).

Guidance on the safety of vitamin D intake is provided by several health agencies that released tolerable upper intake levels (ULs) for vitamin D as shown in Table 2. The IOM and EFSA have both set their UL for vitamin D at 100 μg (4,000 IU) per day for adults (45, 46, 78). Given an individual recommendation (e.g., RDA or equivalent) of 10–20 μg (400–800 IU), the safety range is 80–90 μg (3,200–3,600 IU) and the safety factor (UL/RDA) is 5–10. The EFSA report on ULs, after reviewing the literature, concluded that a daily dose of 250 μg (10,000 IU) is considered to reflect a “no observed adverse effect level (NOAEL)” in adults because clinical studies evaluating such doses reported no vitamin D toxicity. Furthermore, this NOAEL seems to be biologically sound because the maximum endogenous vitamin D synthesis by natural sun (UV-B) exposure increases 25(OH)D levels equivalent to oral vitamin D intakes of about 500 μg (20,000 IU) daily (92). In view of some uncertainties around this NOAEL an uncertainty factor of 2.5 was chosen leading to an UL of 100 μg (4,000 IU) for adults. The concept of vitamin D safety also consists of the idea of adequate circulating 25(OH)D concentrations as well as those leading to toxicity. There is, however, uncertainty at which concentrations hypercalcemia occurs although it is frequently quoted that hypercalcemia usually only occurs at serum 25(OH)D concentrations above 375–500 nmol/L. Importantly, the IOM has classified circulating 25(OH)D concentrations of 50–125 nmol/L as adequate and concentrations greater than 125 nmol/L, if sustained, as potentially harmful, although this level is far lower than the serum 25(OH)D concentrations associated with hypercalcemia of approximately greater than 375–500 nmol/L. The considerations regarding the term “potentially harmful” for serum 25(OH)D concentrations above 125 nmol/L until those concentrations leading to hypercalcemia is based on some observational studies indicating increased risk of adverse outcomes such as mortality at high 25(OH)D concentrations. It is important to underline that risk of adverse events at 25(OH)D concentrations above 125 nmol/L has only been inconsistently reported in observational studies and the question of causality is still not answered. However, some RCTs seem to support the cautious approach of the IOM since daily vitamin D supplement doses of 100 μg (4,000 IU) or high bolus doses of vitamin D leading to serum 25(OH)D concentrations >125 nmol/L might in specific population groups adversely impact musculo-skeletal and cardiovascular health (77, 78). On the other hand, several other studies using high doses of vitamin D or studying individuals with very high 25(OH)D concentrations did not report on adverse effects (77, 78, 91). Nevertheless, considering these safety issues and some uncertainty regarding the long term effect of high 25(OH)D concentrations, integrated quantitative risk–benefit assessments according to proposed frameworks are warranted (93–96).

**Table 2:** Tolerable upper intake levels for vitamin D.

**Country (health authority)**	**USA and Canada (IOM)**	**Europe (EFSA)**
**Age group**	**Vitamin D in** μ**g per day (1** μ**g** = **40 international units)**	
0–6 months	25	25
6–12 months	37.5	25
1–3 years	62.5	50
4–8 years	75	50
9–10 years	100	50
11–17 years	100	100
18 years and older	100	100
Pregnancy	100	100
Lactation	100	100

*IOM, Institute of Medicine; EFSA, European Food Safety Authority*.

Although the risk of achieving potentially harmful circulating 25(OH)D concentrations by food fortification with vitamin D is likely to be small in the general population, the problem of idiopathic hypercalcemia should not be neglected. A biallelic mutation in the gene encoding for the vitamin D catabolizing enzyme 24-hydroxylase (*CYP24A1*) can cause infantile idiopathic hypercalcemia (97, 98). This mutation results in vitamin D hypersensitivity and may have a prevalence of 1:33,000 births in Europe (98). The health consequences of this mutation in the adolescent and adult population are currently not known. When discussing the safety of vitamin D food fortification it must also be noted that improvement of vitamin D status by systematic food fortification may also likewise decrease the prevalence of persons taking vitamin D supplements exceeding the UL. In this context, it should also be noted that intermittent high dose vitamin D supplementation is quite common but may pose risk of adverse events. While daily vitamin D supplements with doses according to the RDA or equivalents are safe, intermittent high dose vitamin D supplementation may even increase the risk of fractures and falls (90). In this context, we believe that systematic vitamin D food fortification with subsequent improvement of vitamin D status in the general population may likewise decrease the potential public health burden (and costs) associated with overuse/overdosing of vitamin D supplements.

## History of Vitamin D food fortification

Even before vitamin D was discovered, it had been observed that cod liver oil protects against rickets. Interestingly, it has been empirically shown that one teaspoon of cod liver oil, that contains approximately 10 μg (400 IU) of vitamin D per day, is effective in preventing rickets (5). Successful treatment of rickets has also been demonstrated by sunlight or UV exposure of children in the 1920s followed by documentation that irradiation of food such as milk increased its anti-rachitic activity. Vitamin D food fortification has been widely introduced in the 1930s and 1940s in the United States and many other industrialized countries such Great Britain when it became possible to add purified vitamin D itself to food (92). In particular vitamin D fortified milk was produced at that time, but vitamin D has also been added to a variety of foods and beverages including amongst others beer, hot dogs and custard. This food fortification policy was extremely effective in preventing rickets but in the 1950s there was a change in public health policy as food fortification was banned in Great Britain and many other European countries because cases of hypercalcemia were observed that had been suspected to be attributable to vitamin D intoxication. Whether this was really the case is not clear. Beyond the combined effect of vitamin D overdosing due to different sources [heavy vitamin D enrichment of dried milk powder plus vitamin D fortified cereals plus daily supplement with 17.5–20 μg (700–800 IU) of vitamin D] it has also been hypothesized that the hypercalcemic children in Great Britain may have had an inherited disease called Williams syndrome. This syndrome is, apart from other pathologies, associated with hypercalcemia. Unfortunately, methods for measuring circulating 25(OH)D were not available at that time. Some symptoms of hypercalcemia had, however, been observed in infants in the former German Democratic Republic, where infants were supplemented with intermittent doses of 15 mg (600,000 IU) of vitamin D as an effort to prevent rickets (77, 78). In these infants, serum 25(OH)D concentrations increased up to several hundred nmol/L.

## Current Vitamin D food fortification policies

Overviews of current food fortification policies have been reviewed elsewhere (62, 73, 74, 99–104). There is a huge variation in availability of vitamin D fortified food or food with vitamin D bioaddition across the countries. In general, there are mandatory and voluntary vitamin D food fortification policies but their differentiation is not always trivial as there can be varying pressure and implementation success of voluntary vitamin D food fortification. In Finland, for example, the Ministry of Trade and Industry recommended vitamin D fortification of fluid milks, margarines/fat spreads in 2003 on a voluntary, and not mandatory, basis, but most companies complied with the option to fortify resulting in a systematic (mass) vitamin D fortification (105–111). Many other countries allow voluntary vitamin D food fortification but with only insufficient effects on vitamin D intakes at population level (104, 112). Legislation is, of course, the basis for vitamin D food fortification and while we cannot discuss this issue in detail, we want to point out that the general regulation of voluntary food fortification is harmonized across the European Union (104, 113, 114). Several countries, however, still refer to national laws restricting addition of vitamins and minerals to food. In Germany, for example, addition of vitamin D to food is limited to margarine, based on a law of 1942.

As the experience with systematic (mass) vitamin D food fortification in the US, Canada and Finland may provide important guidance for health authorities in other regions, we list the main vitamin D fortified foods currently practiced in these countries in Table 3 (99, 100, 105–111, 115–117).

**Table 3:** Vitamin D food fortification in the United States, Canada and Finland.

**Food (serving)**	**United States**	**Canada**	**Finland**
**VITAMIN D PER SERVING IN** μ**g (1** μ**g** = **40 INTERNATIONAL UNITS)**			
	**Mass fortification (usually mandatory)**		
Fluid cow's milk (250 ml or 1 cup)	2.5–5.0^†^	2.5–5.0^††^	2.5
Margarine/Fat spread (10 g)		1.5–3.0^††^	2.0
	**Fortification of selected brands**		
Yogurt	1.5–5.0 per 170 g	1.0 per 100 g	0.5–1.0 per 100 g
Cheese slice (16 g)	1.5		
Orange juice (125 ml or 1/2 cup)	1.25	1.25	1.25
Plant-based milk such as soy, oat or almond (250 ml or 1 cup)	1.5–3.0	1.5–3.0	1.9–3.75
Margarine 10 g	0.75–5.0		
Bread (100 g)	2.25		1.7
Cereals, ready-to-eat (1/2–3/4 cup)	1–2.5	1.0	3.0 per 100 g

† *FDA in 2016 permitted voluntary “doubling” of mandatory vitamin D in milk*.

†† *Health Canada will require doubling of mandatory amounts by 2020*.

In particular, the example of Finland can serve as a benchmark for future vitamin D food fortification policies in other countries. In Finland, vitamin D status has recently been assessed in nationally representative samples before and after introduction of systematic vitamin D food fortification (105). These results are based on Vitamin D Standardization Program (VDSP)-standardized 25(OH)D data (105), whereas older Finnish reports without VDSP data should only be interpreted with caution (106–110). In 2003, a systematic voluntary food fortification was introduced in Finland with the recommendation to add vitamin D at a dose of 10 μg/100 g to all fat spreads and at a dose of 0.5 μg/100 g to all fluid milk products. In 2010, these fortification recommendations were doubled to 20 μg/100 g in all fat spreads and 1.0 μg/100 g in all fluid milk products. In a nationally representative survey of Finnish adults, changes in serum 25(OH)D concentrations from 2000 to 2011 were investigated (105). Mean serum 25(OH)D concentrations increased from 47.6 nmol/L in the year 2000 to 65.4 nmol/L in 2011. The prevalence of 25(OH)D concentrations below 30, 40, and 50 nmol/L, respectively, was 13.0, 32.0, and 55.7% in 2000, and decreased to 0.6, 3.2, and 9.1%, respectively, in 2011. Importantly, serum 25(OH)D concentrations increased from 2000 to 2011 by about 34 nmol/L in individuals with 25(OH)D concentrations <30 nmol/L in 2000, whereas there was only an increase of about 11 nmol/L in individuals with 25(OH)D concentrations ≥50 nmol/L in 2000. In 2011, only 8 out of 4051 individuals had serum 25(OH)D concentrations ≥125 nmol/L and of these 8 individuals, 7 were vitamin D supplement users. Although food fortification policy in Finland clearly improved vitamin D status over time, it must be mentioned that there was also an increase in vitamin D supplement use from 11% in 2000 to 41% in 2011. Furthermore, a part of the 25(OH)D increase (approximately 10 nmol/L increase) from 2000 to 2011 cannot be explained by vitamin D fortification and increased use of vitamin D supplements. It is also worth mentioning that fat spreads were already a substantial source of vitamin D intake in 2000 as they were recommended to be fortified by 5–10 μg/ 100 g before systematic fortification started in 2003. Nevertheless, contribution to dietary vitamin D intake from fluid milk products, fat spreads, and fish changed from 4, 9, and 57%, respectively, in 2000 to 34, 10, and 38%, respectively, in 2011. When restricting the analyses to individuals with no supplement use, the mean overall increase in serum 25(OH)D from 2000 to 2011 was 6 nmol/L higher in individuals who consumed fluid milks products as compared to those who did not.

Therefore, and to conclude, the Finnish vitamin D nutrition policy, based on appropriate simulations, has considerably improved vitamin D status in the general Finnish population. This implementation of a systematic vitamin D food fortification programme respresents an example of a successful public health action that may inform similar approaches in other countries. Importantly, vitamin D food fortification policies have been re-evaluated and modified if necessary (105, 118). Apart from Western countries, there are also efforts for vitamin D food fortification in countries such as India (with e.g., vitamin D fortified milk), Jordan (with e.g., vitamin D fortified bread) and several others (73, 119–121).

## Modeling to inform strategies for Vitamin D food fortification

Identifying the need for systematic vitamin D food fortification requires, of course, the assessment of 25(OH)D status and vitamin D intakes in a respective country or population in order to show that the dietary vitamin D requirements are not met. These data, which should at best be derived from a nationally representative sample of the population, can then serve as the basis for modeling vitamin D food fortification scenarios to meet the vitamin D requirements (122–135). A particular focus on groups at highest risk of profound vitamin D deficiency (e.g., those in high-risk ethnic groups or with restrictive diets) is also important.

Apart from the excellent “real-life” data from Finland on the effect of systematic vitamin D food fortification, there exist of course mathematical models to estimate different scenarios of vitamin D food fortification on vitamin D intakes and 25(OH)D status for a given population or country. In a very simplified view there are three different approaches for modeling effects of vitamin D food fortification scenarios. First, based on vitamin D intakes and nutrition habits in the population it can be estimated how vitamin D fortification affects nutritional vitamin D intakes by simply adding existing and additional vitamin D intakes by food fortification (112). Second, based on the previous approach and the availability of 25(OH)D concentrations and by use of a dose-response equation of vitamin D intake and 25(OH)D serum concentrations, it can be estimated how vitamin D fortification affects not only dietary vitamin D intakes but also serum 25(OH)D concentrations (127). Third, in addition to the second approach the additional impact of UV exposure with its seasonal variation is considered to model the effect of vitamin D food fortification on 25(OH)D serum concentrations (125, 126, 128). All of these models have their limitations in particular due to some underlying assumptions so that cautious interpretation of the results is warranted. Regarding underlying assumptions it is important to note that when analyzing data from RCTs on vitamin D food fortification, it has been calculated that for every 1 μg (40 IU) ingested vitamin D, the serum 25(OH)D concentration increases by 1.2 nmol/L (95% confidence interval 0.72–1.68 nmol/L) (123). Simple modeling of an equation on the vitamin D intake-serum 25(OH)D relationship does, however, not reflect potentially modifying factors such as body mass index, age, basal serum 25(OH)D concentrations or genetics (136).

## Cost-effectiveness of Vitamin D food fortification

When considering introduction of systematic vitamin D food fortification, a key question relates to whether or not such a public health intervention is likely to be cost-effective (137–151). In general, micronutrient fortification is considered as being one of the most cost-effective public health interventions (137). With reference to vitamin D food fortification there is, however, only limited evidence available on its cost-effectiveness. Nevertheless, the available studies on this issue point toward the notion that systematic vitamin D fortification (or vitamin D supplementation) may indeed be highly cost-effective (137–153). Regarding the costs for a typical food fortification programme, Fiedler et al. estimated the following distribution of costs: 80% recurrent production costs, 8% marketing and education costs, 7% food control and monitoring costs, and 5% other programme-specific recurrent production costs (137). Using these cost distributions and obtaining annual costs for 20 μg (800 IU) vitamin D per day of 0.11 Euros per person and annual costs for 200 mg calcium per day of 0.22 Euros per person it was estimated by Sandmann et al. that the implementation of a vitamin D plus calcium fortification programme in Germany would cost 41 million Euros per year while saving 365 million Euros per year as a result of reduced fracture costs (139). This would translate into a benefit-cost ratio of 9:1 which is even more conservative than other estimates of the cost-effectiveness of pure vitamin D interventions with even higher benefit-cost ratios (138, 140–149). We are well aware that more data are needed on the cost-effectiveness of systematic vitamin D fortification but we conclude that, despite limited evidence, the available literature suggests that this approach is highly likely to be cost-effective. Despite these promising data, it must be stressed that the overall general health impact of systematic vitamin D food fortification or supplementation can only roughly be estimated (154–156). It should also be mentioned that most studies assessed the cost-effectiveness of vitamin D food fortification in the elderly population and not in the whole population. Beyond cost effectiveness it is, however of course, extremely important that such fortification approaches are also well perceived and accepted by the population itself. This seems to be the case for vitamin D as e.g., shown by a study in Germany (152). The Finnish data also suggest that vitamin D food fortification is well accepted and fortified foods are considered part of the habitual diet (105).

## Suggestions for Vitamin D food fortification

**Figure 2:**
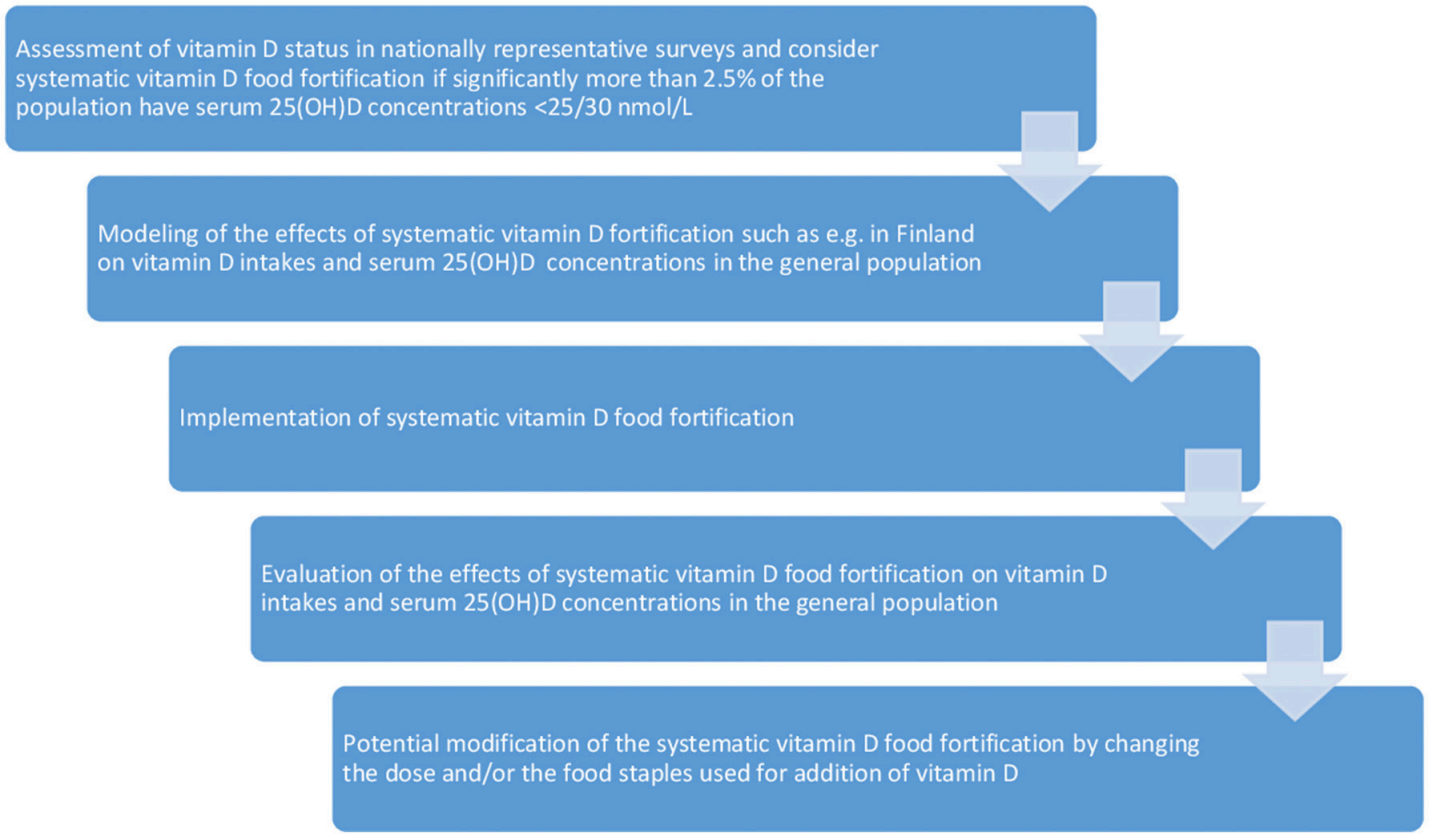
Guidance for vitamin D food fortification.

There is definitely no clear answer on how to implement systematic vitamin D food fortification in countries where the vitamin D dietary requirements are not met by a significant part of the general population. Nevertheless, we want to provide some guidance for this task (see Figure 2).

A first step is, of course, the evaluation of the vitamin D status and intakes from nationally representative nutrition and health surveys. Definition of a precise goal for vitamin D food fortification is not a trivial task, but the general aim is, of course, to improve vitamin D status while avoiding or minimizing risk of potential toxicity related to overdosing of vitamin D. The Guidelines on food fortification with micronutrients by the World Health organization (WHO) define the goal of food fortification as follows: “to provide most (97.5%) of individuals in the population group(s) at greatest risk of deficiency with an adequate intake of specific micronutrients, without causing a risk of excessive intakes in this or other groups” (153). The WHO guideline defines inadequate intakes as intakes below the EAR, which corresponds to a serum 25(OH)D concentration of 40 nmol/L according to the IOM report. Although not clearly outlined in the WHO guideline it appears reasonable to argue that intakes below this goal are a reason for public health actions. Being aware of the heterogeneity of nutritional vitamin D guidelines we are convinced that if significantly more than 2.5% of the population have 25(OH)D concentrations below 25–30 nmol/L there is a justified need for public health interventions including vitamin D food fortification, which becomes imperative if the prevalence increases close to or exceeds 20% in either the entire population or in populations subgroups.

Regarding the goal of vitamin D food fortification we are well aware that in the IOM report, the RDA for vitamin D intakes corresponds to 50 nmol/L of serum 25(OH)D, and one may ask why we should not aim for this level in almost (97.5%) the entire population. Bringing almost everyone to a level of at least 50 nmol/L of serum 25(OH)D is, however, considered unrealistic, costly, ineffective and (in particular) potentially risky because this means that the target median intake would need to be set at very high levels (153, 157). It must be considered that even at vitamin D intakes between the EAR and the RDA and respective serum 25(OH)D concentrations of 40–50 nmol/L, the majority of the individuals would meet their dietary vitamin D requirements. The WHO guideline on food fortification suggests the target is to shift the intake distribution upwards so that only 2.5% of the population have an intake below the EAR (154). Thus, nearly everyone in the population should have a daily vitamin D intake of at least about 10 μg (400 IU) per day. This would, for a hypothetical usual intake distribution, result in a target median intake about 1.5 times above the RDA and approximately 20% of the population would have intakes below the RDA (153). This hypothetical example does, however, not fully apply for vitamin D because the distribution of 25(OH)D is different as shown in Finland where achieving a mean serum 25(OH)D concentration of 65 nmol/L by systematic vitamin D food fortification was sufficient to decrease the prevalence of 25(OH)D concentrations <30 nmol/L in the general population below 1% (105). This provides extremely strong arguments for the safety of vitamin D because the Finish data indicate that with vitamin D food fortification there is a much higher increase in 25(OH)D in those with very low 25(OH)D concentrations at baseline when compared to those with high 25(OH)D concentrations at baseline.

In general, we see two broad approaches to implementation of systematic vitamin D food fortification. The first one adheres to previous systematic vitamin D food fortifications in countries with similar population characteristics in terms of vitamin D status and food habits that have been evaluated with regard to safety and efficacy, as it has been done in Finland. It is a reasonable approach to follow the example of the Finnish vitamin D food fortification policy when modeling of the effects of such a vitamin D food fortification results in a significant and safe improvement of vitamin D status and intakes. The second approach is based on an “optimal modeling” of systematic vitamin D food fortification of many different food products to increase vitamin D status. It is clear from hypothetical models of vitamin D intakes and status that fortifying multiple food staples is desirable because such approaches reach broader parts of the population and are theoretically more safe than just fortifying one or a few food staples. However, such approaches are more costly and will likewise have a lower acceptance as there are currently no countries using and evaluating such approaches.

As some of the authors reside in Austria and Germany we wish to briefly outline the conceivable food fortification scenarios in these two countries (158–168). Data from national representative samples on vitamin D status and intakes in Austria and Germany are shown in Table 4. It is obvious from the high prevalence of low 25(OH)D concentrations and the low dietary vitamin D intakes that vitamin D food fortification is necessary in these countries to meet the vitamin D requirements. In this context, we wish to underline that there is long experience with vitamin D food fortification of dairy products in different countries covering a wide range of different nutritional habits, lifestyle and latitudes. Therefore, we are of the opinion that systematic vitamin D fortification of milk and margarine/fat spreads (dairy products) according to the approach used in Finland would be a reasonable approach also in Austria and Germany. Modeling of such a vitamin D fortification scenario on vitamin D intakes and on serum 25(OH)D concentrations is, of course, definitely required. Importantly, in Austria and Germany there is a similar yet slightly lower dairy intake but slightly higher 25(OH)D concentrations compared to Finland before the introduction of systematic vitamin D food fortification, suggesting that the Finnish approach may be a good model for these two countries (169). High prevalences of vitamin D deficiency with a need for improvement of vitamin D status are, however, also observed in many other European countries such as Poland with 16% of the general population having serum 25(OH)D concentrations below 25 nmol/L (170, 171).

**Table 4:** Vitamin D intakes and status in Austria and Germany.

**Group**	**Intakes in** μ**g per day**		**Serum/plasma 25-hydroxyvitamin D in nmol/L or percentages below a 25-hydroxyvitamin D cut-off concentration**						
	**Mean (SD)**	**Median (25th to75th percentile or IQR)**	**Mean (SD)**	**Median (25th to75th percentile or IQR)**	<**25**	<**30**	<**40**	<**50**	<**75**
			**nmol/L**		**Percentages**				
**AUSTRIA**									
**Austrian Study on Nutritional Status 2017**									
Female adults	2.3 (2.4)	1.7 (1.1–2.8)							
Male adults	2.7 (2.6)	2.0 (1.2–3.4)							
**Austrian Study on Nutritional Status 2012**									
Girls 7–14 years		1.26 (1.00)		44.9 (32.5)	22.3			62.3	
Boys 7–14 years		1.39 (0.93)		44.7 (36.0)	17.7			55.8	
Women 18–64 years		2.6 (2.2–3.1)		57.4 (47.5)	11.6			39.8	
Women 65–80 years		3.2 (2.5–3.8)		42.3 (28.5)	19.9			42.4	
Men 18–64 years		3.9 (3.1–4.7)		55.9 (51.2)	14.2			43.9	
Men 65–80 years		3.9 (2.9–5.0)		41.8 (28.4)	20.4			44.4	
**GERMANY**									
**German Health Interview and Examination Survey for Children (KiGGS; 2003 until 2006)**									
All children			54.0 (19.2)	52.9 (39.4–71.6)	6.0	12.5	25.9	45.6	83.8
Girls 6–11 years		1.3 (0.8–2.1)							
Girls 12–17 years		1.7 (1.2–2.5)							
Boys 6–11 years		1.4 (0.9–2.1)							
Boys 12–17 years		2.2 (1.5–3.3)							
**German Health Interview and Examination Survey for Adults (DEGS1; 2008 until 2011)**									
All adults			50.1 (18.1)	47.7 (36.1–60.8)	4.2	15.2	34.3	56.0	90.9
**German National Health Interview and Examination Survey (GNHIES; 1997 until 1999)**									
Women		2.31 (1.53–3.56)							
Men		2.81 (1.89–4.44)							

In general, following the examples of other countries with vitamin D fortification of milk and margarines/fat spreads may likewise facilitate the implementation and the acceptance of mass fortification in the population. Additionally or alternatively, fortification of other foods such as bread may be considered in particular if dietary vitamin D requirements cannot be adequately met with vitamin D fortification of milk and margarine/fat spreads. Standardized measurements of 25(OH)D status and assessment of overall vitamin D intakes in nationally representative samples before and after implementation of vitamin D food fortification should be, of course, a condition sine qua non. While we hope for and work on the improvement of food fortification approaches in the future, it is now time to take action and work on the improvement of vitamin D status in countries where significant parts of the population fail to meet the dietary requirements. For this aim, the best evaluated vitamin D food fortification strategies with the likewise highest rates of successful implementation should be pursued.

## Conclusions

In this review, we outlined the background, rationale and current status of systematic vitamin D food fortification and also gave some guidance for implementation of such an approach (see Table 5 for key points). We are of the opinion that the huge gap between the nutritional vitamin D guideline recommendations and the high prevalence of individuals who do not meet their vitamin D requirements calls for public health actions that can be performed by systematic vitamin D food fortification. While there are still many questions surrounding this issue, several countries do have long experience with systematic vitamin D food fortification (172–176). The successful and well evaluated real-life experience with the Finnish food fortification policy may be used as a benchmark for other countries with similar population characteristics. We do hope that our work helps to introduce and modify vitamin D food fortification in those countries where it is needed in order to prevent the significant public health burden of vitamin D deficiency and its adverse consequences.

**Table 5:** Key points.

*Health authorities recommend target serum 25(OH)D concentrations ranging from ≥25 to ≥50 nmol/L (≥10 to ≥20 ng/mL),
corresponding to a daily vitamin D intake of 10–20 μg (400–800 IU)
*Most populations fail to meet these recommended dietary vitamin D requirements
*Systematic vitamin D food fortification is an effective and safe approach to improve vitamin D status in the general population
*Some countries such as the US, Canada, India and Finland have already introduced systematic vitamin D food fortification
*Introduction and/or modification of systematic vitamin D food fortification is required in many countries to improve public health, and should be based on modeling scenarios and efficacy data of vitamin D food fortification from other countries such as Finland

## Author contributions

SP, WM, and AZ drafted an initial version of the manuscript. All authors contributed to manuscript revision, read and approved the submitted version.

### Conflict of interest statement

The authors declare that the research was conducted in the absence of any commercial or financial relationships that could be construed as a potential conflict of interest.
